# Supplementary material for: Interface Engineering of ZnO-Decorated ZnFe2O4 for Enhanced CO2 Reduction Performance
Source: Molecules. 2025 Oct 4;30(19):3980. doi: 10.3390/molecules30193980 (PMC12526438; doi:10.3390/molecules30193980)
Supplement: Supplementary file 1 [file molecules-30-03980-s001.zip › molecules-3866635-supplementary.pdf]

Supporting Information

Interface Engineering of ZnO-Decorated $ZnFe_2O_4$ for Enhanced CO_2 Reduction Performance

Congyu Cai [1], Yufeng Sun [2], Yulan Xiao [1], Weiye Zheng [1], Minhui Pan [1] and Weiwei Wang [1,*]

1 School of Life Science and Chemistry, Minnan Science and Technology College, Quanzhou 362332, China; caicongyu@mku.edu.cn (C.C.); pan-minhui@mku.edu.cn (M.P.)

2 School of Humanities, Minnan Science and Technology College, Quanzhou 362332, China; sunyufeng@mku.edu.cn

* Correspondence: wangweiwei@mku.edu.cn

S3. Experimental section

S3.1 Materials

The chemicals used in this work include zinc nitrate hexahydrate ($Zn(NO_3)_2 \cdot 6H_2O$, A.R.), Iron(III) nitrate nonahydrate ($Fe(NO_3)_3 \cdot 9H_2O$, A.R.)，Polyethylene glycol (PEG-6000, A.R.)，Ethylene glycol (EG, A.R.)，Urea ($CO(NH_2)_2$, A.R.)，Oxalic acid dihydrate ($H_2C_2O_4 \cdot 2H_2O$, A.R.)，Sodium acetate (CH_3COONa, A.R.)，Cetyltrimethylammonium bromide (CTAB, A.R.)，Sodium hydroxide (NaOH, A.R.) were purchased from Aladdin reagent Co., Ltd. In this experiment, all chemicals were utilized as received without any other treatment. Ultrapure water was used throughout all experiments.

S3.2 Preparation of $ZnFe_2O_4$ and ZnO Microspheres via Solvothermal Method

At room temperature, polyethylene glycol (PEG-6000, 2.5 g) was dissolved in ethylene glycol (50 mL) under magnetic stirring until complete dissolution. Subsequently, zinc nitrate hexahydrate ($Zn(NO_3)_2 \cdot 6H_2O$, 1 mmol) and iron(III) nitrate nonahydrate ($Fe(NO_3)_3 \cdot 9H_2O$, 2 mmol) sequentially to the solution with continuous stirring until full dissolution. Then, urea (0.15 g) and oxalic acid ($H_2C_2O_4 \cdot 2H_2O$, 0.15 g) to the mixture. The resulting solution was stirred vigorously for 60 minutes at room temperature. Followed by the addition of 50 mL of cetyltrimethylammonium bromide (CTAB, 10 g/L). After stirring for an additional 30 minutes to achieve homogeneity. Transfer the homogeneous mixture into a 100 mL Teflon-lined stainless-steel autoclave. Seal the autoclave and heat it in a preheated oven at 200 °C for 24 hours. After the reaction, allow the autoclave to cool naturally to room temperature. Collect the precipitate by centrifugation, and wash it thoroughly with deionized water and absolute ethanol alternately several times to remove residual ions and organics. Dry the washed product in a vacuum oven at 100 °C for 8 hours to obtain the $ZnFe_2O_4$ microspheres.

At room temperature, polyethylene glycol (PEG-6000, 2.5 g) was dissolved in ethylene glycol (50 mL) under magnetic stirring until complete dissolution. Subsequently, $Zn(NO_3)_2 \cdot 6H_2O$ (1 mmol) was added and stirred until fully dissolved, followed by the addition of urea (0.15 g) and $H_2C_2O_4 \cdot 2H_2O$ (0.15 g). The mixture was stirred vigorously for 60 minutes. Then, 20 mL of NaOH solution (40 g/L) was slowly introduced under continuous stirring, and 50 mL of cetyltrimethylammonium bromide (CTAB, 10 g/L) was added thereafter. After stirring for another 30 minutes to achieve a homogeneous mixture, the resulting solution was transferred into a Teflon-lined stainless-steel autoclave and heated at 200 °C for 24 h. Upon natural cooling to room temperature, the precipitate was collected by centrifugation, washed alternately with deionized water and absolute ethanol several times, and dried at 100 °C for 8 hours under ambient atmosphere to obtain ZnO microspheres.

S3.3 Preparation of ZnO/$ZnFe_2O_4$ Composite via Solvothermal Method

Solution A: Dissolve polyethylene glycol (PEG-6000, 2.5 g) in ethylene glycol (50 mL) under magnetic stirring at room temperature until complete dissolution. Sequentially add

$Zn(NO_3)_2{\cdot}6H_2O$ (1.5 mmol) and $Fe(NO_3)_3{\cdot}9H_2O$ (2 mmol), stirring until dissolved. Then add urea (0.15 g) and $H_2C_2O_4{\cdot}2H_2O$ (0.15 g). Stir the mixture vigorously for 60 minutes at room temperature.

Solution B: Dissolve sodium hydroxide (NaOH, 0.8 g) in deionized water (20 mL) under stirring. Slowly pour Solution B into Solution A under continuous stirring to form Solution C. Gradually add cetyltrimethylammonium bromide (10g/L CTAB, 50mL) to Solution C under vigorous stirring. Continue stirring until a homogeneous mixture is obtained for an additional 30 minutes. Transfer the final mixture into a Teflon-lined stainless-steel autoclave. Seal the autoclave and heat it in a preheated oven at 200 °C for 24 hours. After the reaction, allow the autoclave to cool naturally to room temperature. Collect the precipitate by centrifugation, and wash it thoroughly with deionized water and absolute ethanol alternately several times. Dry the washed product in an oven at 100 °C for 8 hours, under ambient atmosphere to obtain the $ZnO/ZnFe_2O_4$ composite.

S3.4 Catalysts characterization

The morphological characteristics of the samples were examined by scanning electron microscopy (SEM, Thermo Fisher Apreo S) and transmission electron microscope (TEM, FEI Tecnai Spirit). The crystalline structure was characterized using X-ray diffraction (XRD, Thermo Scientific ESCALAB 250) with Cu Kα radiation. X-ray photoelectron spectroscopy (XPS) analysis was performed on a Thermo Fisher Scientific K-Alpha spectrometer using a monochromatic Al Kα X-ray source. Textural properties, including specific surface area and pore size distribution, were determined by low-temperature (77 K) nitrogen adsorption-desorption measurements, with data interpreted using the Brunauer-Emmett-Teller (BET) theory. Electron paramagnetic resonance (EPR, Bruker MS5000) technique was employed to detect and analyze free radicals or species with unpaired electrons in the samples. The optical absorption properties were evaluated by ultraviolet-visible diffuse reflectance spectroscopy (UV-vis DRS) using a Shimadzu UV-3600 spectrophotometer. The photoluminescence (PL) spectra were acquired at room temperature using a HORIBA Fluoromax-4 spectrofluorometer.

The working electrode was fabricated as follows: 10.0 mg catalyst was added into 200.0 μL of a mixture of ethanol and Nafion solution (v/v = 18/2) via sonication for 30 min. Subsequently, 0.3 mL as-prepared mixture was cast onto the surface of an FTO substrate (1.0 cm × 1.0 cm) and dried at room temperature for 2 h.

Photoelectrochemical characters of as-prepared samples were investigated on an electrochemical workstation (CHI660E) with a three-electrode system. In this system, a photocatalyst-coated FTO glass as the working electrode, a Pt plate as the counter electrode, an Ag/AgCl electrode as the reference electrode, and 0.1 M Na_2SO_4 aqueous solution (pH = 7) as the electrolyte. Electrochemical impedance spectroscopy (EIS) and chronoamperometry (I-t) curves were measured on a CHI660E electrochemical workstation. M-S curves were tested at 1000 Hz under light conditions. For the EIS test, the working electrode was tested over the frequency range of 0.1 to 100,000 Hz at 0 V. The I - t curves were measured under the irradiation of a 300 W xenon

lamp (Beijing Perfectlight PLS-SXE 300 C), with the light source alternately turned on and off for 50 s during the test. For in situ DRIFTS, the dynamic evolution process was monitored using a Fourier in-situ infrared spectrometer (Nicoletis10, Thermo Fisher) at different time points. Prior to measurement, the samples were purged with N_2 for 20 min in the sample chamber to eliminate surface contaminants and achieve stabilization. After collecting the background spectrum at t = 0 min, which was subsequently subtracted from all measurements, the gas source was switched to CO_2: N_2 mixture (2:8 v/v) with simultaneous light illumination (300W xenon lamp). Samples were collected every 2 minutes for a total duration of 30 minutes.

S3.5 Photocatalytic CO_2 reduction reaction

The photocatalytic reduction of CO_2 is carried out in a stainless steel reactor with a light-transmitting and high-pressure resistant quartz glass at the top (thickness: 10 mm; diameter: 50 mm; pressure rating: 1.5 MPa), a heating device at the bottom, a thermocouple inside the reactor (accuracy: ±0.5 °C), and a sample stage for the catalyst. The light source for the photocatalytic reaction test was a 300W xenon lamp ((300 W Xe lamp with visible-light filter, λ ⩾ 420 nm) PLS-SXE 300 C, Beijing Perfectlight, Beijing, China). For the experiment, 20 mg of catalyst powder was evenly spread in the reactor tray and placed in the reactor perpendicular to the beam (The distance between the lamp and the reactor surface was fixed at 2 cm). The system temperature was raised to and maintained at 120 °C by a PID temperature controller. The reactor was sealed, pressurized with high-purity CO_2 (99.999%) to 0.5 MPa to check for leaks, and then evacuated. Subsequently, 0.3 mL of deionized water was injected into the reactor as the proton source. The light source was then turned on to initiate the reaction, which lasted for 6 h. Gas products were automatically sampled and analyzed every hour using an online gas chromatograph (GC9790II PLF-01, HP-MOLESIEVE, 30 m × 0.53 mm × 25 μm, China) equipped with a methanizer and connected to both a thermal conductivity detector (TCD) and a flame ionization detector (FID). High-purity Ar (99.999%) was used as the carrier gas. The GC was calibrated using standard gas mixtures of known concentrations (CO, CH_4, CO_2) before and during the experimental series to ensure quantitative accuracy. The detection limit for CH_4 and CO is approximately 0.1 ppm.

S3.6 XRD

The average crystallite size (D) of the nanoparticles was estimated using the Debye–Scherrer equation:

$$D = K\lambda / (\beta\cos\theta)$$

where K is the Scherrer constant (0.89), λ is the wavelength of the X-ray radiation (0.15406 nm), β is the full width at half maximum (FWHM) of the diffraction peak (in radians), and θ is the Bragg diffraction angle (in degrees).

Based on this equation, the average crystallite size and lattice parameters of the samples were calculated from the XRD diffraction data.

Tab.S1 XRD structural parameter of samples $ZnFe_2O_4$ and $ZnO/ZnFe_2O_4$.

Photocatalytics	Peak position (nm)	Area	Height	FWHM	Variable	Standard card
$ZnFe_2O_4$	35.3°	617.6	455	0.84	1	JCPDS No. 79-1105
$ZnO/ZnFe_2O_4$	35.3°	2670.4	6506	0.27	4.3	JCPDS No. 89-7102

Tab.S2 Textural property of samples $ZnFe_2O_4$ and $ZnO/ZnFe_2O_4$.

Samples	S_{BET} m^2/g	V_P cm^3/g	d_P nm
$ZnFe_2O_4$	115.97	0.137	4.73
$ZnO/ZnFe_2O_4$	11.76	0.098	33.44

Tab.S3 XPS analysis of Zn and Fe

Samples	Atomic/%		$ZnO/ZnFe_2O_4$: $ZnFe_2O_4$	
	Zn(2p3/2)	Fe(2p3/2)	ΔE_{Zn}:(Zn/65)	ΔE_{Fe}: (Fe/56)
$ZnFe_2O_4$	19.05	3.03	4.78	14.79
$ZnO/ZnFe_2O_4$	27.97	1.00	3.25	44.8
			0.7:1	3.0:1

Tab.S4 PL of Relative Signal Amplitude Comparison

Samples	Peak Height (a.u.)	Normalized Area
$ZnFe_2O_4$	188.42	1323.53
$ZnO/ZnFe_2O_4$	152.60	1123.47

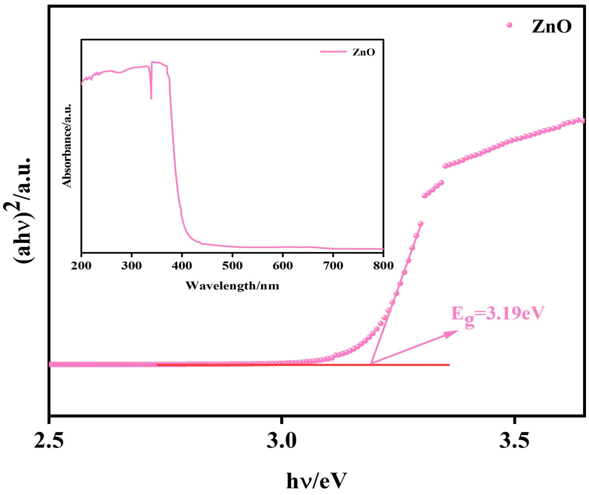

Figure.S1 UV-vis DRS.

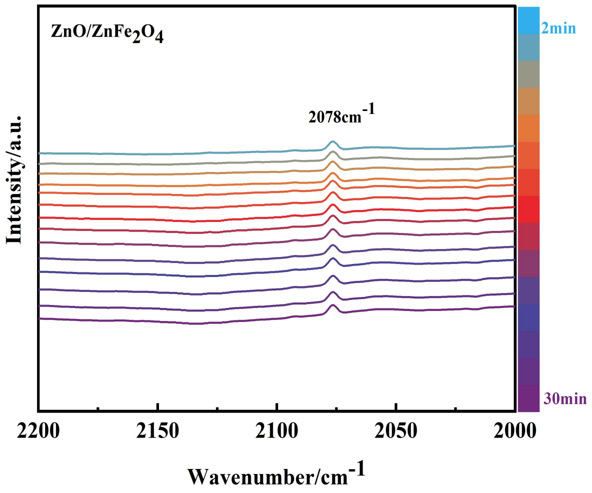

Figure.S2 In-situ FTIR infrared local magnification image.

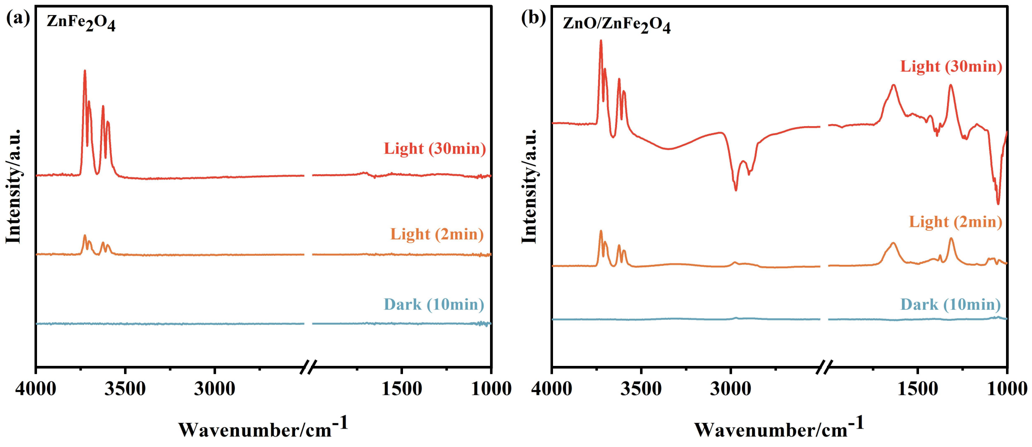

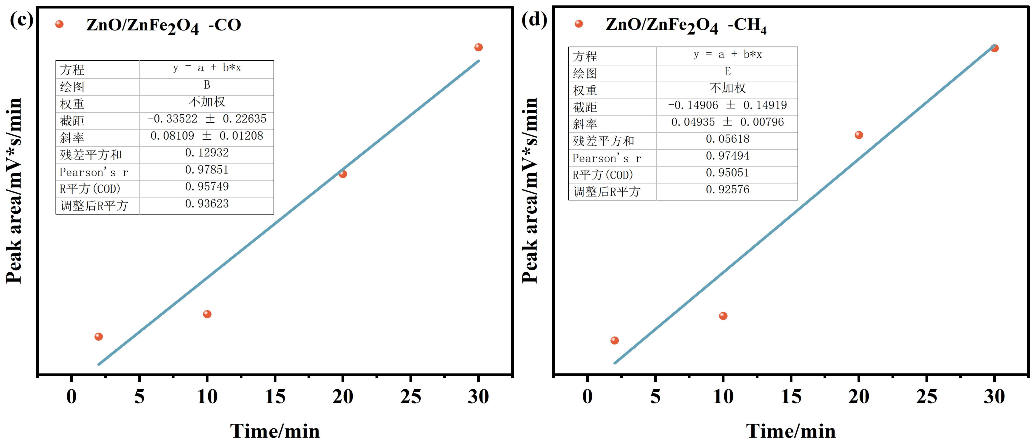

Figure.S3 In-situ FTIR spectra of (a)$ZnFe_2O_4$ and (b)$ZnO/ZnFe_2O_4$ for the photocatalytic CO_2 reduction, (c) CO and (d) CH_4 for rate constants.

.

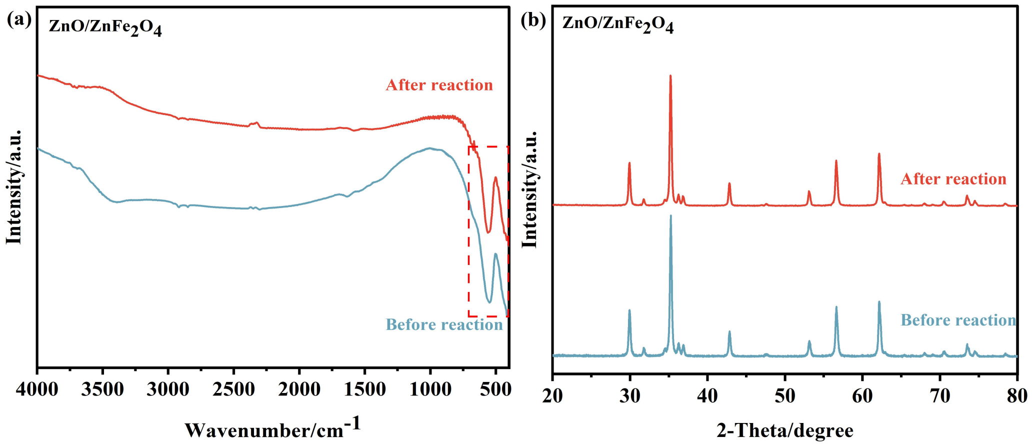

Figure S4. (a) FTIR spectra, (b) XRD patterns of $ZnO/ZnFe_2O_4$ before and after photocatalytic reactions.
